# High-temperature high-pressure microfluidic system for rapid screening of supercritical CO_2_ foaming agents

**DOI:** 10.1038/s41598-021-82839-4

**Published:** 2021-02-09

**Authors:** Ayrat Gizzatov, Scott Pierobon, Zuhair AlYousef, Guoqing Jian, Xingyu Fan, Ali Abedini, Amr I. Abdel-Fattah

**Affiliations:** 1 Aramco Services Company, Aramco Research Center-Boston, 400 Technology Square, Cambridge, MA 02139 USA; 2 Interface Fluidics Ltd., National Institute for Nanotechnology, 11421 Saskatchewan Dr NW #4-087, Edmonton, AB T6G 2M9 Canada; 3 EXPEC ARC, Reservoir Engineering Technology Division, Saudi Aramco, Dhahran, 31311 Saudi Arabia; 4 Present Address: Pacific Northwest National Laboratory, Richland, WA 99352 USA

**Keywords:** Carbon capture and storage, Chemistry, Energy science and technology, Engineering, Nanoscience and technology

## Abstract

CO_2_ foam helps to increase the viscosity of CO_2_ flood fluid and thus improve the process efficiency of the anthropogenic greenhouse gas’s subsurface utilization and sequestration. Successful CO_2_ foam formation mandates the development of high-performance chemicals at close to reservoir conditions, which in turn requires extensive laboratory tests and evaluations. This work demonstrates the utilization of a microfluidic reservoir analogue for rapid evaluation and screening of commercial surfactants (i.e., Cocamidopropyl Hydroxysultaine, Lauramidopropyl Betaine, Tallow Amine Ethoxylate, N,N,N′ Trimethyl-N′-Tallow-1,3-diaminopropane, and Sodium Alpha Olefin Sulfonate) based on their performance to produce supercritical CO_2_ foam at high salinity, temperature, and pressure conditions. The microfluidic analogue was designed to represent the pore sizes of the geologic reservoir rock and to operate at 100 °C and 13.8 MPa. Values of the pressure drop across the microfluidic analogue during flow of the CO_2_ foam through its pore network was used to evaluate the strength of the generated foam and utilized only milliliters of liquid. The transparent microfluidic pore network allows in-situ quantitative visualization of CO_2_ foam to calculate its half-life under static conditions while observing if there is any damage to the pore network due to precipitation and blockage. The microfluidic mobility reduction results agree with those of foam loop rheometer measurements, however, the microfluidic approach provided more accurate foam stability data to differentiate the foaming agent as compared with conventional balk testing. The results obtained here supports the utility of microfluidic systems for rapid screening of chemicals for carbon sequestration or enhanced oil recovery operations.

## Introduction

Emission of anthropogenic CO_2_ into the atmosphere is a major global environmental stressor. Reducing global emissions is a significant challenge, and the most viable solution requires unilateral cooperation between governments, industries, and research communities. Reduction of CO_2_ emission can be possible through two broad approaches. The first approach substitutes high carbon emission technologies with those of lower or no carbon footprints^1^. This approach mainly seeks the decommissioning of coal and liquid fossil fuel power plants and swapping them with energy plants operating with natural gas, nuclear, hydroelectricity, solar, wind, etc^2,3^. A similar technological revolution is taking place in the electrification of cars to reduce emissions from internal combustion engines^4–6^. The second approach is carbon capture, utilization, and storage technologies, at commercial scale. Capturing the waste CO_2_ from large sources (e.g., power plants, cement and metal smelting factories) has been implemented using several methods (e.g., post-combustion capture and oxyfuel combustion capture)^7,8^. Coupled with the capture approach, the utilization of CO_2_ as a basis material for high-value products is encouraging. However, the conversion efficacy and up-scaling both need to be further optimized^9,10^. Carbon sequestration, on the other hand, is considered one of the most promising approaches. It involves the storage of large amounts of CO_2_ in underground geological formations—mainly oil reservoirs, abandoned gas fields, and deep saline aquifers^11,12^. CO_2_ injection into depleted oil reservoirs for the purpose of CO_2_ enhanced oil recovery (EOR) is a more common approach that ultimately lowers the carbon footprint of fossil fuel emissions to support continuing energy demands^13^.

A major challenge to carbon sequestration and CO_2_-based EOR in deep formations is the very low viscosity and density of CO_2_ (~ 0.1–0.5 mPa s and ~ 0.56 g/cm^3^) compared to water (0.3–1 mPa s and ~ 0.99 g/cm^3^) or conventional crude oil (0.6–10 mPa s and ~ 0.8–0.9 g/cm^3^). Low CO_2_ viscosity results in unfavorable mobility ratio, poor conformance, and fingering of the CO_2_ front, leading to early breakthrough and bypassing vertical and/or lateral portions of the formation and thus potential hydrocarbon resources. Lower density, on the other hand, leads to CO_2_ gravity override and bypassing of deeper portions of the reservoir, leading to ineffective sweep efficiencies for carbon sequestration and low reservoir productivity in EOR operations. The aforementioned deficiencies are more pronounced in hydrocarbon recovery processes in reservoir formations comprised of different layers with diverse permeabilities. In these oil reservoirs, the CO_2_ takes preferential pathways, flowing through more permeable zones without contacting oil in lower permeability zones^14,15^. Theoretically speaking, mitigating these deficiencies requires increasing the density and viscosity of the supercritical CO_2_. Increasing the density of supercritical CO_2_ phase, e.g., through the addition of chemicals, is not technically feasible. Increasing its viscosity, on the other hand, is more feasible and can be achieved by either the addition of chemical thickeners directly into the CO_2_ phase^16^ or forming CO_2_ foams via the addition of surfactants into the aqueous phase in the porous structure of the reservoir rock^14^. Foaming the injected CO_2_ by injecting very dilute amounts of surfactant solutions with the CO_2_ phase has the potential to significantly increase the sequestration efficiency. The foamed CO_2_ has a larger apparent viscosity and traps more of the CO_2_ in situ, reducing the overall CO_2_ mobility. Increasing the trapped CO_2_ means that CO_2_ comes in contact with the resident saline water for longer periods of time and hence, will increase the amount of dissolved CO_2_ in the brine.

Developing chemical formulations to produce CO_2_ foam for reservoir applications has been investigated and reported in several publications^14,15,17–21^. Reservoirs around the world have different properties (e.g., mineralogy, depth, temperature, and water salinity), necessitating a wide range of formulation chemistries to be developed for specific reservoir conditions. Development of efficient formulations is an expensive process, requiring numerous trials and extensive tests performed at in-situ reservoir pressure and temperature conditions while changing other experimental variables. Core flooding and foam loop rheometer testing are the most common methods applied to screen formulation performance. While these methods provide insights into formulation performance, they suffer from long technician times—from days to weeks—and generally require large volumes of fluid samples. The cost associated with these conventional testing methods motivates the development of rapid and accurate performance screening of formulations at relevant reservoir conditions.

Microfluidics technology has provided significant benefits in research and industry across various fields, with a growing track of applications in industrial fluids and chemistries^22–28^. There have also been precedent applications of microfluidics-based methods in CO_2_ and energy sectors^23–25,29–33^. Microfluidic systems can visualize the performance of pore-scale interactions of fluids at relevant operating conditions while requiring only a few milliliters of fluid samples^34–36^. Various fabrication methods^22,27^ provide a unique opportunity to develop microfluidic chips with desired channel shapes, dimensions, and surfaces, to improve mixing of phases, or to better represent the pore structure of reservoir rock^37^. While developing microfluidics for experiments at higher temperatures (> 100 °C) and pressures (> 7 MPa) combined with high-resolution optical access is a challenging process, technical progress has enabled some high-temperature high-pressure microfluidics experiments^38–41^.

This work demonstrates the utilization of a microfluidic reservoir analogue and presents an approach to rapidly screen and evaluate CO_2_ foam formulations at high-temperatures (up to 100 °C) and high-pressure (up to 13.8 MPa) conditions. The analogue consists of a porous medium representing a reservoir with relevant pore geometries. Six potential foam formulations were tested with CO_2_ at different concentrations and qualities through measurement of pressure drop, and the resulting mobility reduction factor. Bright-field microscopy combined with an in-house developed image analysis algorithm is employed to visualize the foam, count the foam lamellae, and determine the foam half-life. Finally, the obtained microfluidic test results presented here are compared with those of conventional widely accepted bulk foam testing techniques.

## Experimental

### Materials

**Figure 1:**
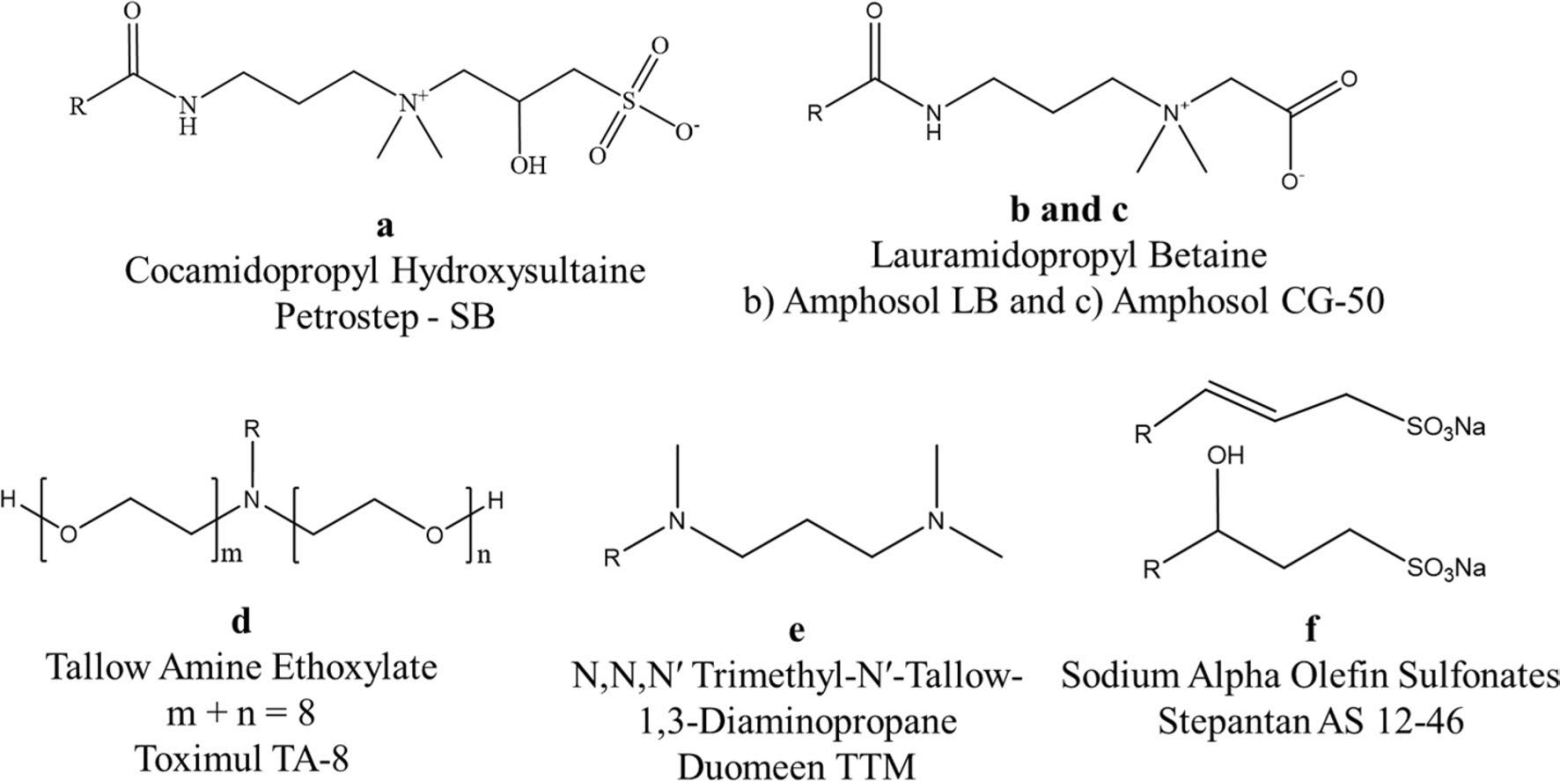
Chemical structures of the surfactants used in this study. (**a**) Cocamidopropyl hydroxysultaine used as active ingredient in Petrostep SB, (**b**, **c**) lauramidopropyl betaine with R = 11–13 in (**b**) Amphosol LB (from narrow cut methyl esters) and R = coco in (**c**) Amphosol CG-50 (derived from refined coconut oil), (**d**) tallow amine ethoxylate with m + n = 8 and R =  ~ 19 in Toximul TA-8, (**e**) N,N,N′ trimethyl-N′-tallow-1,3-diaminopropane with R = 16–18 in Duomeen TTM, and (**f**) sodium alpha olefin sulfonates with R = 11–13 in Stepantan AS 12-46.

Surfactants used in this study (Fig. 1) were obtained from the following suppliers and used as-is: ~ 44 wt% active Petrostep SB (Cocamidopropyl Hydroxysultaine), ~ 30 wt% active Amphosol LB (Lauramidopropyl Betaine), ~ 37 wt% active Amphosol CG-50 (Cocamidopropyl Betaine), 100 wt% active Toximul TA-8 (Tallow Amine Ethoxylate with approximately eight moles of Ethylene Oxide), and 46 wt.% active Stepantan AS 12-46 (Sodium Alpha Olefin Sulfonate) from Stepan Company (Northfield, USA), 95 wt% active Duomeen TTM (N,N,N′ Trimethyl-N′-Tallow-1,3-Diaminopropane) from AkzoNobel (Amsterdam, The Netherlands). Other chemicals were obtained from VWR international (Radnor, USA).

### Surfactant aqueous solutions

Artificial Brine 1 (~ 57,000 mg/L total dissolved solids (TDS)) and Brine 2 (~ 120,000 mg/L TDS) were prepared by dissolving the salts indicated in Table 1 in 1 L of deionized (DI) water. Surfactants were then dissolved in artificial brines to achieve the necessary wt% concentration of active ingredients. Except Duomeen TTM, which was protonated with 1 M HCl first to allow solubilization in an aqueous phase. Solutions were visually tested for stability/formation of precipitates over 7 days of incubation in an oven at 100 °C in a glass microwave reaction vial with polytetrafluoroethylene lined seal (Chemglass Life Sciences LLC, Vineland, USA). Duomeen TTM’s chemical stability at high-salinity and high-temperature conditions for up to 30 days was confirmed in previous studies utilizing Agilent 1100 high-performance liquid chromatography coupled with a G4218 evaporative light scattering detector^42^.

**Table 1:** Compositions of artificial brines used to prepare surfactant solutions.

Salt	Brine 1 (mol/L)	Brine 1 (g/L)	Brine 2 (mol/L)	Brine 2 (g/L)
NaCl	0.7022	41.04	1.2764	74.59
CaCl_2_·2H_2_O	0.0162	2.39	0.3387	49.79
MgCl_2_·6H_2_O	0.0868	17.65	0.0648	13.17
BaCl_2_	0.00	0	0.0001	0.01
Na_2_SO_4_	0.0447	6.34	0.0042	0.6
NaHCO_3_	0.0020	0.17	0.00607	0.51
Total		67.59		138.67

### Microfluidic testing

**Figure 2:**
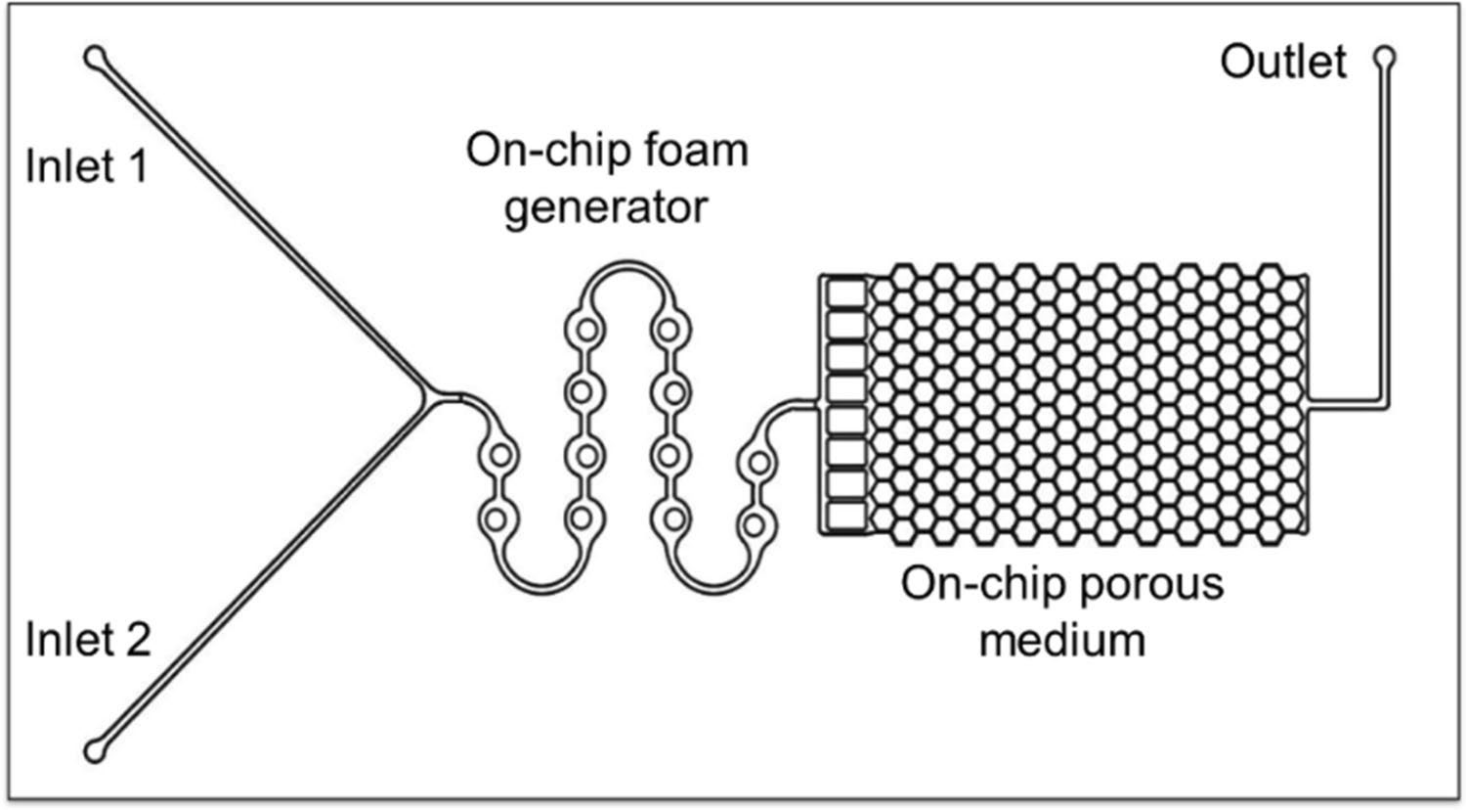
Microfluidic chip pattern designed and developed to study Sc-CO_2_ surfactant foams.

Figure 2 shows the microfluidic chip design used to screen various supercritical CO_2_ (Sc-CO_2_) surfactant foams at different conditions. The chip has one inlet connected to the surfactant solution and another to the CO_2_ injection system. An on-chip foam generator comprising a series of off-centered circular mixing channels was designed to produce consistent and uniform Sc-CO_2_ foams. The relative centers of the circular mixing channel walls of 0.5 mm inner diameter and 1.0 mm outer diameter were radially displaced by 0.05–0.1 mm. The foam generator channel dimensions are 200 µm (width) × 10 µm (depth). The porous medium of the microfluidic chip is a homogeneous hexagonal channel network where the foam behavior at the pore-scale level of the reservoir rock is replicated and studied. The porous medium has pore dimensions of 30 µm (width) × 10 µm (depth), correlating to a hydraulic radius of 15 µm. The pattern was transferred onto a silicon substrate and etched to the specified dimensions using reactive ion etching (RIE). To complete the fabrication, the RIE-etched silicon substrate was anodically bonded to a glass slide to seal off the analogue from the atmosphere, provide a viewing window, and sustain microchannel pressures > 13.8 MPa.

**Figure 3:**
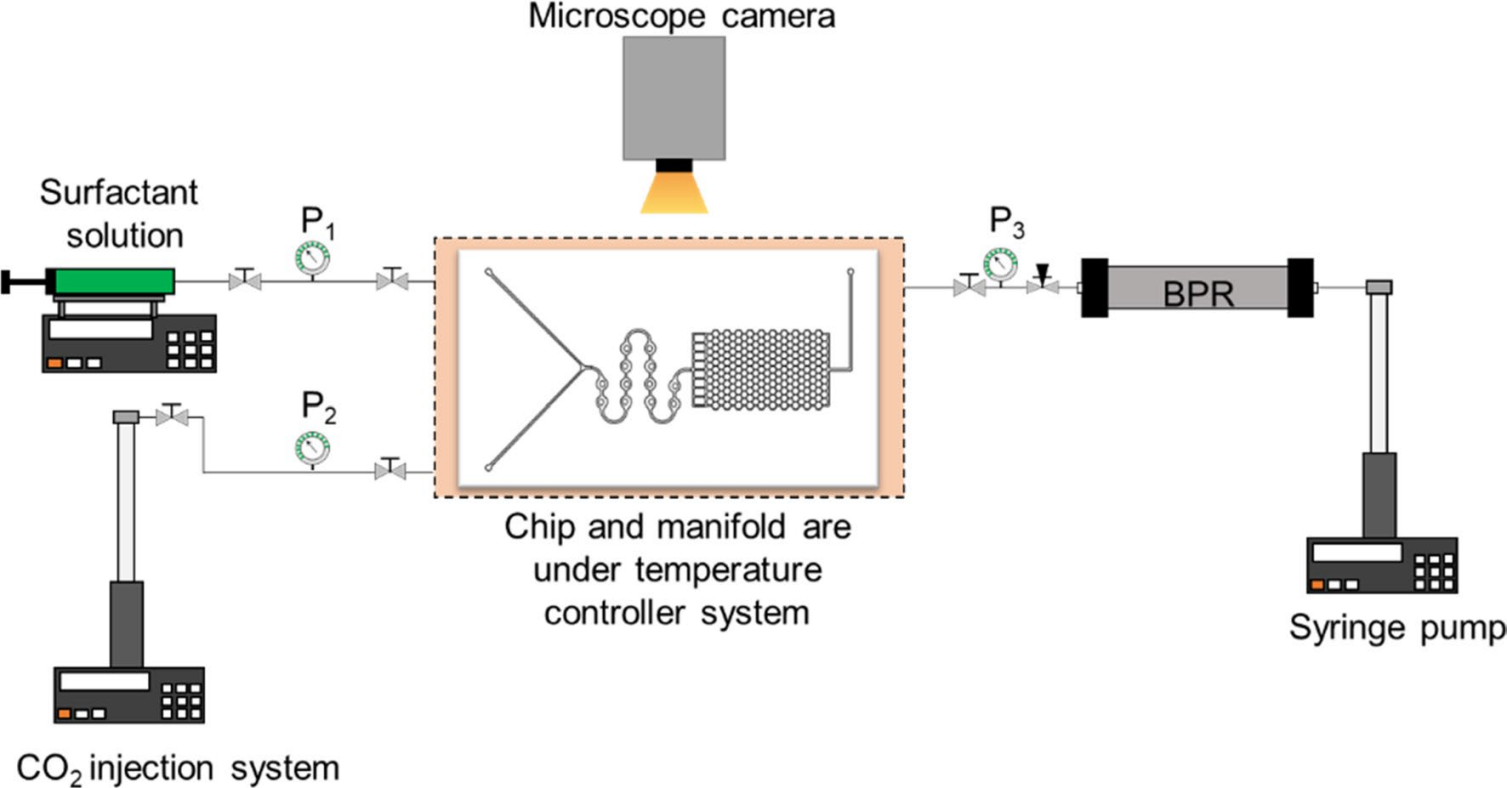
Schematic of the microfluidic setup used to study Sc-CO_2_ surfactant foams at a high-temperature high-pressure condition.

Figure 3 shows the schematic of the experimental setup used to conduct the microfluidic testing. A new microfluidic chip was used for each test to ensure that the initial chip condition was the same for all experiments. The microfluidic chip was mounted onto an in-house designed high-temperature high-pressure manifold and connected to the external experimental system: a syringe pump for surfactant injection (Chemyx Fusion 6000), two pumps for CO_2_ injection and back-pressure regulation of the chip outlet pressure, respectively (Teledyne ISCO 260D), and external high-accuracy pressure gauges (Omega Engineering PX409-3.5KGUSBH). Foam testing was performed at 100 °C, and the back-pressure regulator was maintained at 13.8 MPa during foam injection for all microfluidic experiments to maintain CO_2_ at supercritical conditions. In addition, the entire fluid injection lines from pumps into the microfluidic device was insulated and maintained at the test temperature using a temperature controller (Omega Engineering CNi3222). The pump used to inject the CO_2_ was kept at the test temperature using a temperature-controlled mineral oil bath circulator and an insulation sleeve. It is also noted that due to the high thermal conductivity of the silicon material, the thermal equilibration of fluids occurs quickly and effectively inside the microfluidic chip.

### Phase 1 procedure

Each surfactant solution at the desired concentration was injected with CO_2_ into the microfluidic chip under constant flow control mode from Inlet 1 and 2, respectively. The CO_2_ to surfactant flowrate ratio was adjusted to achieve a specific foam quality during the experiment. Foam quality is defined as the flowrate of CO_2_ divided by the total flowrate (i.e., total of CO_2_ and surfactant solution flowrates). Control tests using pure brine samples with Sc-CO_2_ were also conducted as base cases.

### Phase 2 procedure

Identical to the Phase 1 Procedure with additional equilibrium pressure drop measured across the chip to determine the mobility reduction factor (MRF), which quantifies resistance to CO_2_ flow, as

1 $$MRF = \frac{{{{\Delta P_{{\left( {CO_{2} \;foam} \right)}} } \mathord{\left/ {\vphantom {{\Delta P_{{\left( {CO_{2} \;foam} \right)}} } {Q_{{\left( {CO_{2} \;foam} \right)}} }}} \right. \kern-\nulldelimiterspace} {Q_{{\left( {CO_{2} \;foam} \right)}} }}}}{{{{\Delta P_{{\left( {pure\;CO_{2} } \right)}} } \mathord{\left/ {\vphantom {{\Delta P_{{\left( {pure\;CO_{2} } \right)}} } {Q_{{\left( {pure\;CO_{2} } \right)}} }}} \right. \kern-\nulldelimiterspace} {Q_{{\left( {pure\;CO_{2} } \right)}} }}}}$$

where Q is volumetric flow rate specified by the injection pumps at the test pressure and temperature.

### Phase 3 procedure

To analyze the foam stability as a function of time, the chip was sealed off from pumps after the equilibrium pressure drop was achieved, as described in “Phase 2 procedure”. The experimental setup was identical as for Phase 2 except that the external Omega pressure sensors were replaced with flow-through pressure sensors (DJ Instruments DF2-SS-01-2500). The foam lamellae count and foam half-life were determined through image analysis of time-lapse images of the porous medium. Foam stability analysis in the sealed-off chip was also performed at the test temperature and pressure.

### Bulk supercritical-CO_2_ foam analysis

Bulk Sc-CO_2_ foam height analysis at 23.4 MPa (~ 3394 psi) pressure and 100 °C temperature was performed using Krüss High Pressure Foam Analyzer instrument. Measurements were completed by Eurotechnica GmbH (Bargteheide, Germany). For each measurement, surfactant solution was first loaded into a precleaned cylindrical test cell of diameter of ~ 40 mm with a 100 mm high window. The cell was then heated, partially filled with CO_2_, and left for 24 h to saturate the aqueous phase. Foam was produced by sparging CO_2_ through a sintered glass plate at 50 mL/min into the surfactant solution, with the foam height and rate of decay determined from images captured by an external camera.

### Foam loop rheometer

**Figure 4:**
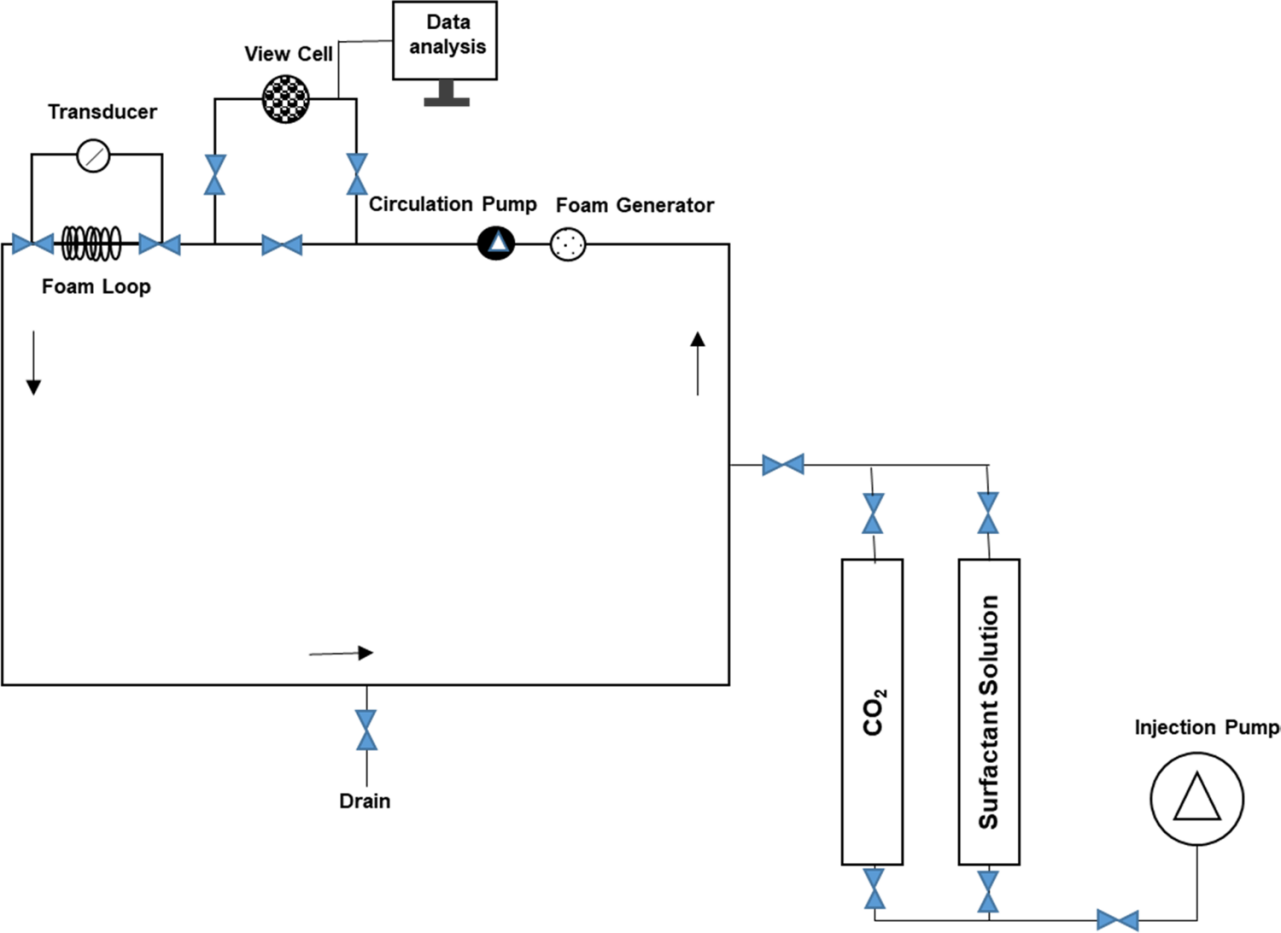
Schematic of the custom-built, high-temperature high-pressure foam loop rheometer.

A custom-made high-temperature high-pressure foam loop rheometer (Fig. 4) was used to evaluate the rheology of Sc-CO_2_ foams at 12 MPa (~ 1740 psi) pressure and 90 °C temperature. The instrument is equipped with a sapphire window cell for visualization and a steel tube loop to measure viscosity under dynamic conditions. Prior to taking measurements, the rheometer was calibrated using non-Newtonian fluid standards. First, the surfactant foaming agent solution (0.50 wt%) was introduced and allowed it to equilibrate in the cell. The Sc-CO_2_ was then introduced and allowed to equilibrate at the desired temperature and pressure. Measurements were performed for Sc-CO_2_ foam qualities at 90%, and shear rates ranging from 10 to 600/s. Foam is a non-Newtonian fluid with apparent viscosity that is shear rate dependent. The apparent viscosity $\left({\mu }_{app}\right)$ of the generated foam was calculated using Eqs. (2)–(4)^43^:

2 $${\mu }_{app}=\frac{\tau }{\gamma }$$

3 $$\tau = \frac{D \Delta P}{4L}$$

4 $$\gamma =\frac{8 }{D}$$

where $\tau$ is a shear stress, $\gamma$ is a shear rate, *D* is the tube diameter, *∆P* is the differential pressure across the loop, *L* is the tube length, and ν is the velocity.

## Results and discussions

The twelve formulations comprised of the six surfactants shown in Fig. 1 dissolved in two different aqueous phases (Brine 1 and Brine 2) were evaluated for the generation of Sc-CO_2_ foams. Prior to the microfluidics experiments, surfactants solutions were tested for physical stability in both brines, exhibiting no sign of visual instability. For reservoir applications and field operations, long term chemical stability, on the order of few months, is required to ensure that formulations do not lose their performance during operations. The stability test procedure can be performed as discussed elsewhere^44^. Microfluidics screening procedures were divided into three distinct phases. In the first phase, the twelve at 0.5 wt% concentration were screened for foamability of the foam by flowing through the microfluidic analogue at different foam qualities. Pressure differences across the analogue were recorded, together with visual qualitative analysis of the generated foams. The second phase was designed to investigate the performance of top formulations selected from the first phase at different concentrations. Similar to the first phase, pressure drops from different foam qualities of four formulations at four different concentrations were recorded to calculate the MRF for each case. The third phase involved counting the lamellae and measuring the foam half-life in the analogue of the four formulations at two different concentrations and foam qualities. Microfluidic results were then compared to bulk foam measurements obtained using the high-pressure foam analyzer and foam loop rheometer instruments.

### Phase 1: Rapid screening of formulations for foamability

**Figure 5:**
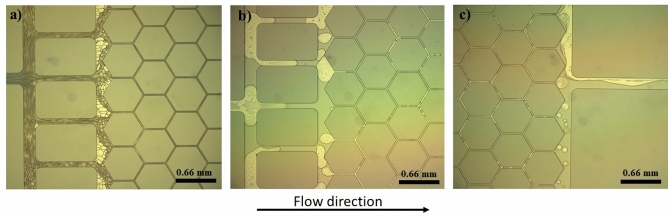
Optical microscope images of the analogue demonstrating examples of the Sc-CO_2_ and aqueous two-phase flows for (**a**) Duomeen TTM, (**b**) Toximul TA-8 both at 0.5 wt% in Brine 1; 70% foam quality and (**c**) CO_2_ and Brine 1 without surfactants; 30% foam quality.

Figure 5 shows the optical microscope images of the analogue during flow of the aqueous and Sc-CO_2_ phases. Figure 5a shows dense foam generated with observable lamellae in the distribution channels at the entrance of the porous medium for Duomeen TTM at 0.5 wt% in Brine 1. Unlike the Duomeen TTM formulation, Fig. 5b reveals that Toximul TA-8 at 0.5 wt% in Brine 1 did not produce a dense foam throughout the analogue and no clearly formed lamellae were observed in the distribution channels. This observation suggests that Duomeen TTM exhibited a better performance in generating dense Sc-CO_2_ foam compared with Toximul TA-under similar testing conditions. Figure 5c depicts the flow of CO_2_ and Brine 1 in the absence of surfactants that resulted in no foam being formed and a laminar co-flow of CO_2_ and brine observed at the outlet of the analogue. High-resolution optical analysis of the foam flow in the analogue provides a rapid tool to qualitatively differentiate formulation performance for generating CO_2_ foam.

**Figure 6:**
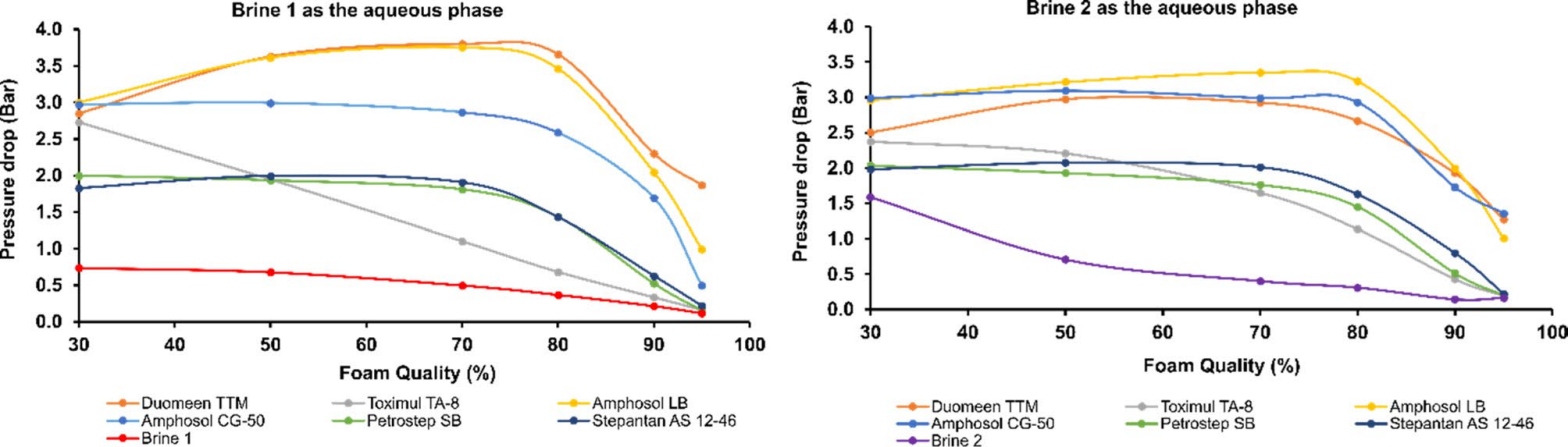
Pressure drop across the microfluidic analogue during injection of the aqueous and Sc-CO_2_ phases. The plot on the left is for Brine 1 and on the right is for Brine 2 once used as an aqueous phase in the presence and absence of surfactants. Concentration of surfactants for all cases is 0.5 wt%.

In addition to optical analysis of the Sc-CO_2_ foam, the pressure drop across the analogue during flow was measured for each test. Higher pressure drop corresponds to reduced mobility as a result of the foam formation. Additionally, smaller bubble size (i.e., higher density of the foam) results in a higher apparent viscosity and thus higher pressure drop. Measurements were performed for multiple foam qualities (30%, 50%, 70%, 80%, 90%, and 95%). In developing the microfluidic test system, the pressure drop of different surfactant formulations varied by < 10% relative error within robust foam quality trends. This repeatability enabled singlicate tests for rapid and accurate screening of surfactants, with test cases re-run only if resulting in trend outliers. Figure 6 shows the pressure drop values recorded for the two-phase flow of CO_2_ and six surfactant formulations at 0.5 wt% concentration both in Brine 1 and Brine 2. Pressure drop measurements were compared and plotted with those of the control cases of CO_2_ with each brine without surfactants. For foam qualities above 80%, the performance of the foam is significantly reduced, resulting in increased mobility of both the CO_2_ and aqueous phases. At these high foam qualities, where the aqueous flow is low and insufficient, there is a lack of lamella to produce high performance foams. In such conditions, flow with the low proportion of aqueous phase resembles the mobility of the gas phase and the pressure drop declines. At 95% foam quality, Toximul TA-8, Petrostep-SB, and Stepantan AS 12-46 exhibited similar performance as Brine 1 and Brine 2 without surfactants, indicating their low performance in forming strong foam with stabilized lamellae. However, Duomeen TTM, Amphosol LB, and Amphosol CG-50 showed better performance in generating stable foams at higher foam qualities. The pressure drop data also demonstrates that these formulations produce foams with different strengths depending on whether Brine 1 or Brine 2 is the aqueous solutions. Specifically, while Toximul TA-8 performed the worst in both brines, it had a much lower pressure drop when used with Brine 1 than with Brine 2. This extreme low efficiency can be attributed to reduced hydration of the hydroxy groups on the amine ethoxylate heads of the surfactant molecules in Brine 1 as compared in Brine 2, leading to higher interfacial tension between the CO_2_ and the aqueous phase in Brine 1. It has been reported that hydration of carbohydrates increases with increasing the concentration of calcium ions (Ca^2+^) in water, especially at high temperatures (i.e., 90 °C)^45^. Considering that Brine 2 contains twenty times more Ca^2+^ ions than Brine 1, similar effects are expected to happen here. Overall, the microfluidic pressure drop data shows that Duomeen TTM, Amphosol LB, and Amphosol CG-50 formulations result in the strongest CO_2_ foams in either brine. The underlying effects of ions in the brines on the performance of specific functional groups of surfactants in CO_2_ foams is an important topic and should be considered for a separate study.

### Phase 2: Sensitivity analysis of the foam to concentration of surfactants

**Figure 7:**
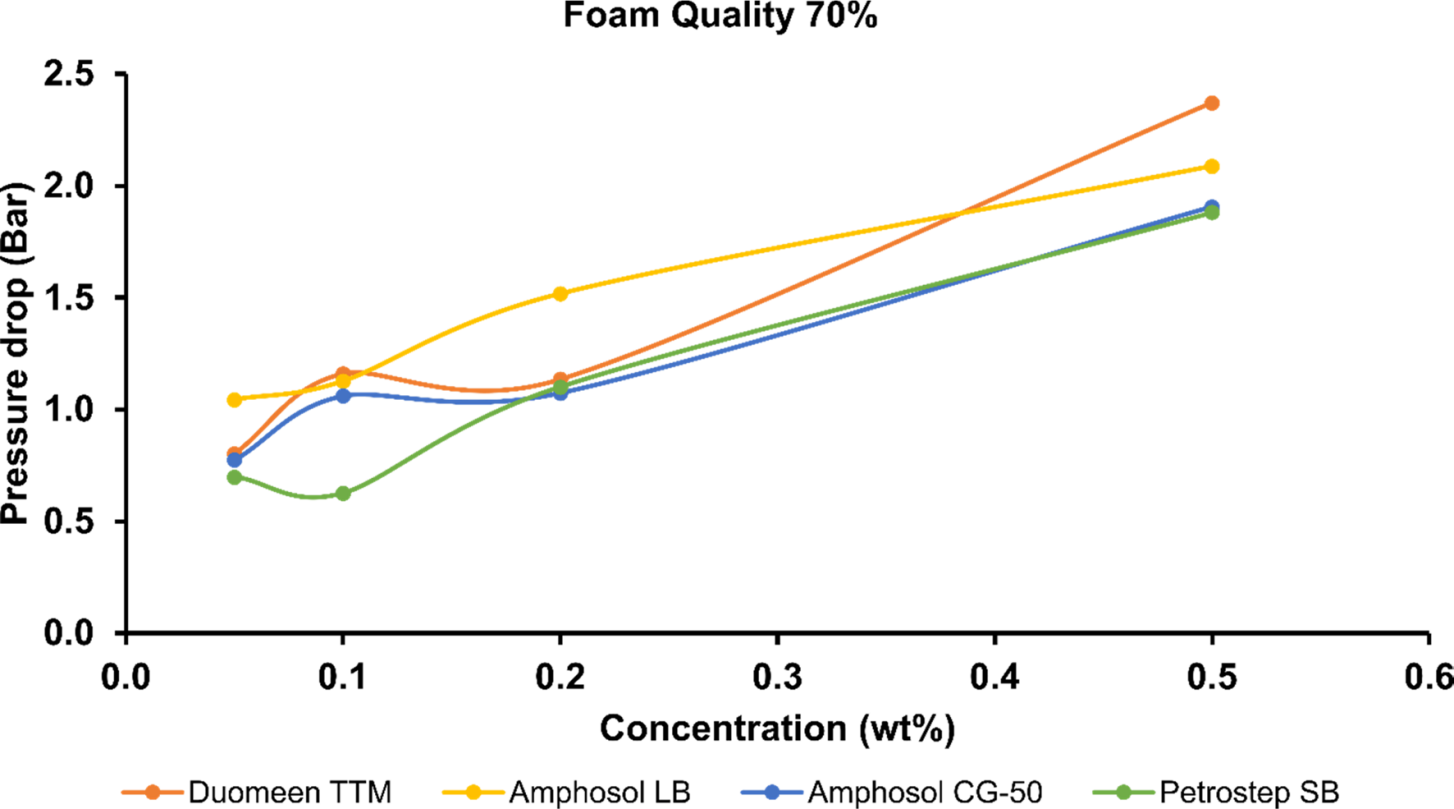
Pressure drop across the microfluidic analogue during flow of the Sc-CO_2_ foam at 0.5, 0.2, 0.1, and 0.05 wt% concentrations and 70% foam quality. Surfactants include Duomeen TTM, Amphosol LB, Amphosol CG-50, and Petrostep SB in Brine 1.

Surfactant concentration in the aqueous phase is a critical parameter in successful and cost-effective implementation of foam injection. Understanding the effect of surfactant concentration on the CO_2_ foam generation process provides insights for more accurate modelling and simulation of pilot tests and field implementation^46,47^. Phase 2 of the microfluidic experiments was aimed to further evaluate the best-performing formulations from Phase 1 in singlicate: Duomeen TTM, Amphosol LB, and Amphosol CG-50. Petrostep SB was also selected for further evaluation due to its zwitterionic chemical structure with favorable stability and transport properties in high-salinity and high-temperature carbonate reservoirs^48^. In Phase 2 of the experiments, CO_2_ foams were evaluated at surfactant concentrations of 0.5, 0.2, 0.1, and 0.05 wt% in Brine 1, at 50%, 70%, and 80% foam qualities. Figure 7 shows the pressure drop of Sc-CO_2_ foam flow with the abovementioned four surfactants at different concentrations at 70% foam quality. As expected, the pressure drop of the foam increases with increased surfactant concentration for all formulations, indicating that higher surfactant concentration results in CO_2_ foams with higher viscosity. The highest pressure drops were achieved with Duomeen TTM and Amphosol LB, and their rank as top performers swapped with increasing concentrations tested. The marginally increased performance of Duomeen TTM at the highest concentration may be due to an optimum surfactant concentration beyond which increased concentration lowers foam stability, as reported for other surfactants^49^. Petrostep SB consistently performed the worst, also in agreement with the results of Phase 1.

**Figure 8:**
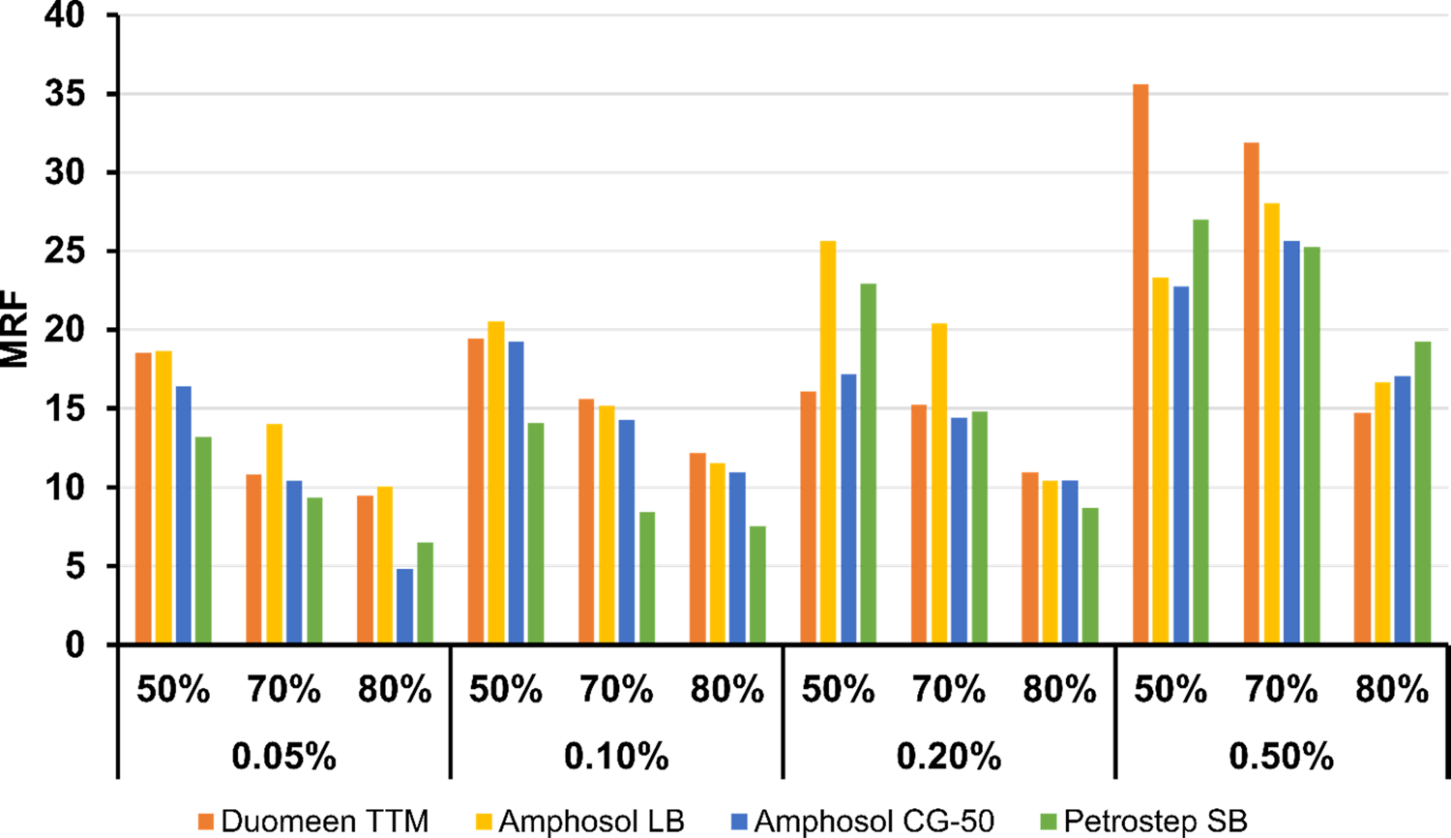
Mobility reduction factors for Sc-CO_2_ foams with surfactants Duomeen TTM, Amphosol LB, Amphosol CG-50, and Petrostep SB, at concentrations of 0.05, 0.1, 0.2, and 0.5 wt% and foam qualities of 50, 70, and 80%.

Figure 8 plots the MRF values for all experiments performed in Phase 2 as functions of surfactant concentration and foam quality. It is observed that surfactants exhibited the lowest MRF values at 80% foam quality, while 50% and 70% foam qualities resulted in higher MRF. Amphosol LB resulted in the highest MRF over all test conditions, with the exceptions of very high MRF with 0.5 wt% Duomeen TTM at 50% and 70% foam quality. Petrostep SB was again the worst performer.

### Phase 3: Half-life of Sc-CO_2_ foam

**Figure 9:**
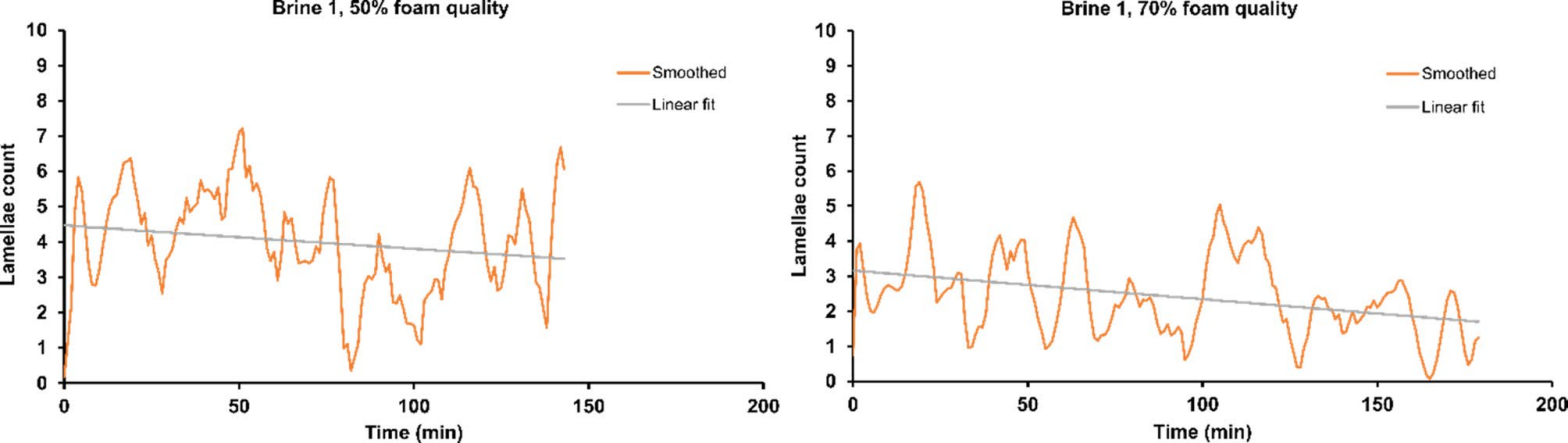
Decay of Savitzky–Golay filtered lamellae counts in the porous media of microfluidic chips during control experiments of sealed-off two-phase flow of Sc-CO_2_ and Brine 1 in the absence of surfactants, at qualities 50% and 70%. Linear fits used to estimate half-life in the absence of exponential decay.

**Figure 10:**
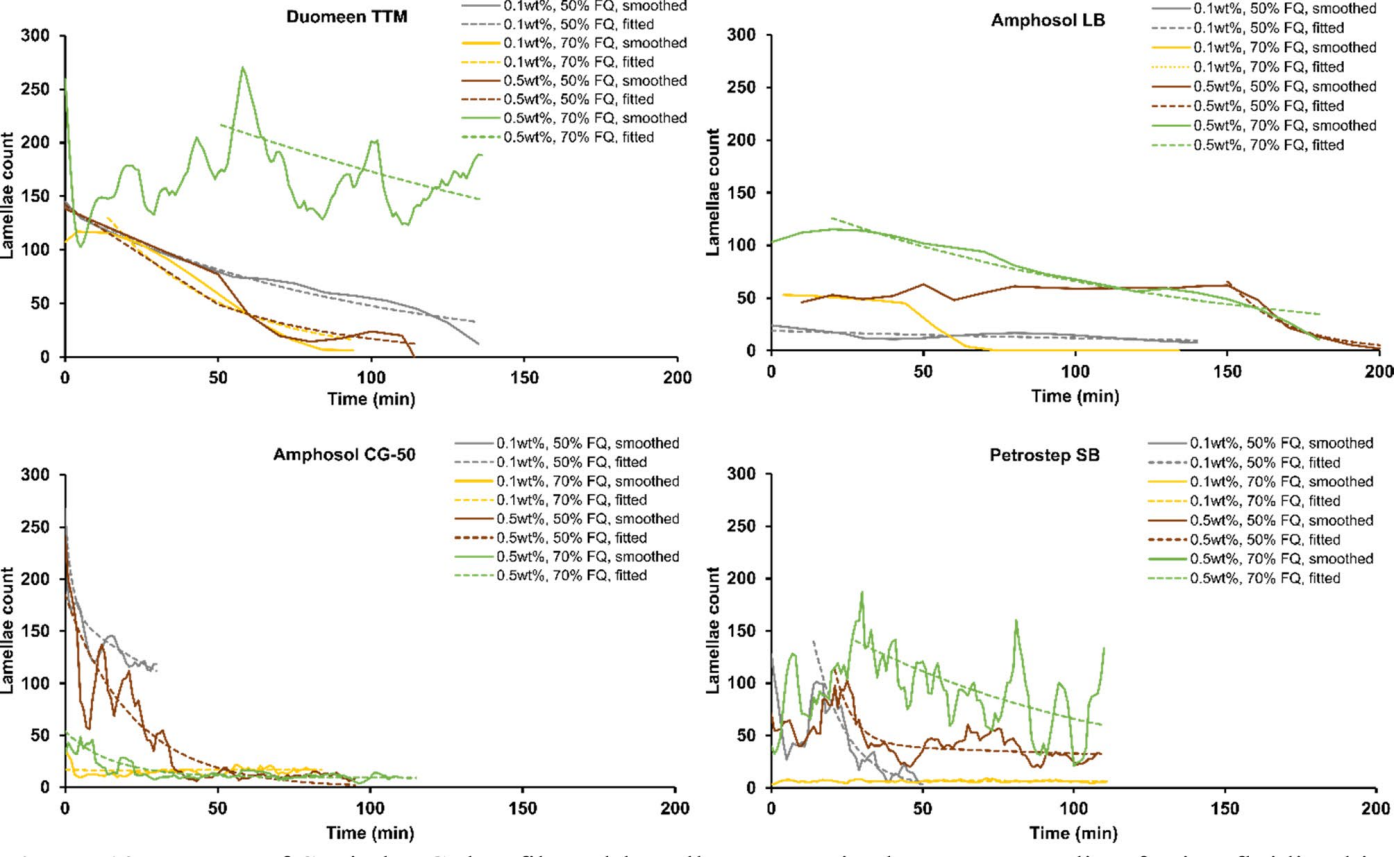
Decay of Savitzky–Golay filtered lamellae counts in the porous media of microfluidic chips during experiments of sealed-off two-phase flow of Sc-CO_2_ with 0.1 wt% and 0.5 wt% surfactants in Brine 1, at qualities 50% and 70%. Exponential fits for half-life calculations are described in the text.

Foam stability can be quantified by its half-life. Conventional foam stability measurements, performed in vertical columns, evaluate the rate of foam collapse through the variation in foam height^50^. However, bulk foam measurements do not fully capture foam stability at the porescale, thus it is not representative of the confined pore space conditions relevant to reservoir rock. Alternatively, microfluidic devices provide high-resolution optical access to foam stability studies at the pore sizes relevant to the reservoirs with tight control over the operating parameters. Table 2 presents foam half-life measurements for 50% and 70% qualities using four formulations at 0.1 and 0.5 wt% concentrations in Brine 1. Half-life time is defined as the period during which the number of foam lamellae decreases to half of its initial number. Figures 9 and 10 show the decay periods of lamellae count from time-zero. The data are presented as Savitzky–Golay smoothed trends for clarity and to reduce stochastic noise (third order polynomial with frame length 11)^51^. To calculate lamellae half-life during each period, an exponential fit in the form of

**Table 2:** Sc-CO_2_ foam half-life determined at 0.1 and 0.5 wt% surfactant concentrations in Brine 1 and at two distinct foam qualities of 50% and 70%.

Formulation	Duomeen TTM				Amphosol LB				Amphosol CG-50				Petrostep SB			
Concentration (wt%)	0.5		0.1		0.5		0.1		0.5		0.1		0.5		0.1	
Foam quality (%)	50	70	50	70	50	70	50	70	50	70	50	70	50	70	50	70
Foam half-life (min)	33	270^a^	64	50	315	127	151	113	15	14	19	1	52	72	36	0

^a^Lower limit to actual half-life calculated from fit of period of highest rate of lamella decrease, which oscillated at 178 ± 35 (mean ± sd) overall.

5 $${\sum }_{n}{a}_{n}{e}^{{b}_{n}x}$$

with two or four coefficients is used (n = 1 or 2)—except for the three trendlines that had fewer than ~ 10 lamellae, for which a linear fit was used to estimate half-life (both controls in Fig. 9, and Petrostep SB 0.1 wt% 70% FQ in Fig. 10). Fits were chosen to minimize root–mean–squared error. Amphosol LB demonstrates the longest half-life stability of the foams, followed by Duomeen TTM. The results are also in agreement with those of Phase 2, where 0.5 wt% concentration of surfactants tend to produce more stable foams when compared to 0.1 wt%. From all three phases discussed, it can be concluded that Amphosol LB and Duomeen TTM are better candidates to generate more stable Sc-CO_2_ foam with higher performance as compared with the rest of the formulations.

### Half-life of Sc-CO_2_ foam in bulk phase

**Figure 11:**
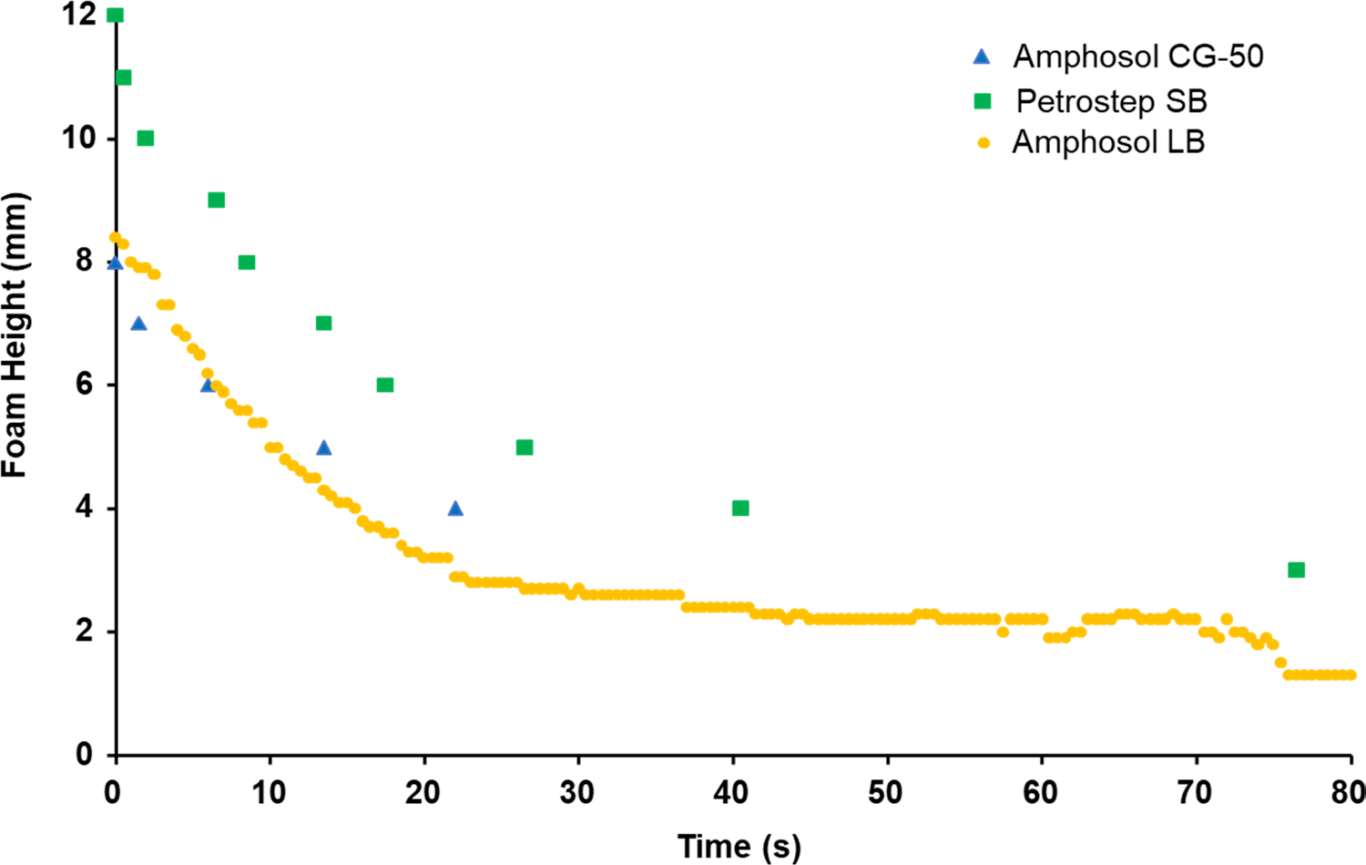
Bulk foam height and stability measurements performed using Krüss High Pressure Foam Analyzer instrument for Sc-CO_2_ and Brine 1 with surfactant concentrations = 0.5 wt%, T = 100 °C, P = 23.4 MPa.

To compare the foam stability results obtained by microfluidic measurements with those of conventional measurement methods, the half-life of Sc-CO_2_ foams were also determined by bulk testing in a vertical column. Foam was produced by purging CO_2_ through the aqueous solution and loaded into a 40 mm diameter column. Control of the foam quality was not feasible compared to that achieved in the microfluidic device. Figure 11 shows the half-life of CO_2_ foams produced using 0.5 wt% Petrostep SB, Amphosol LB, and Amphosol CG-50 in Brine 1. The maximum foam height in a ~ 100 mm height view window was only ~ 10 mm for all three tested formulations. The half-life was less than 20 s for all cases, inconsistent with the long half-life obtained from the microfluidic stability testing (e.g., the half-life of the foam for Amphosol LB in the microfluidic analogue was hours). The bulk CO_2_ foam stability test results revealed that conventional bulk testing is not able to provide reliable and differentiative data, mostly due to the lack of tight control on CO_2_ flow and absence of the porous geometries required for these measurements and relevant to reservoir formations.

### Conventional foam loop rheometer measurements

**Figure 12:**
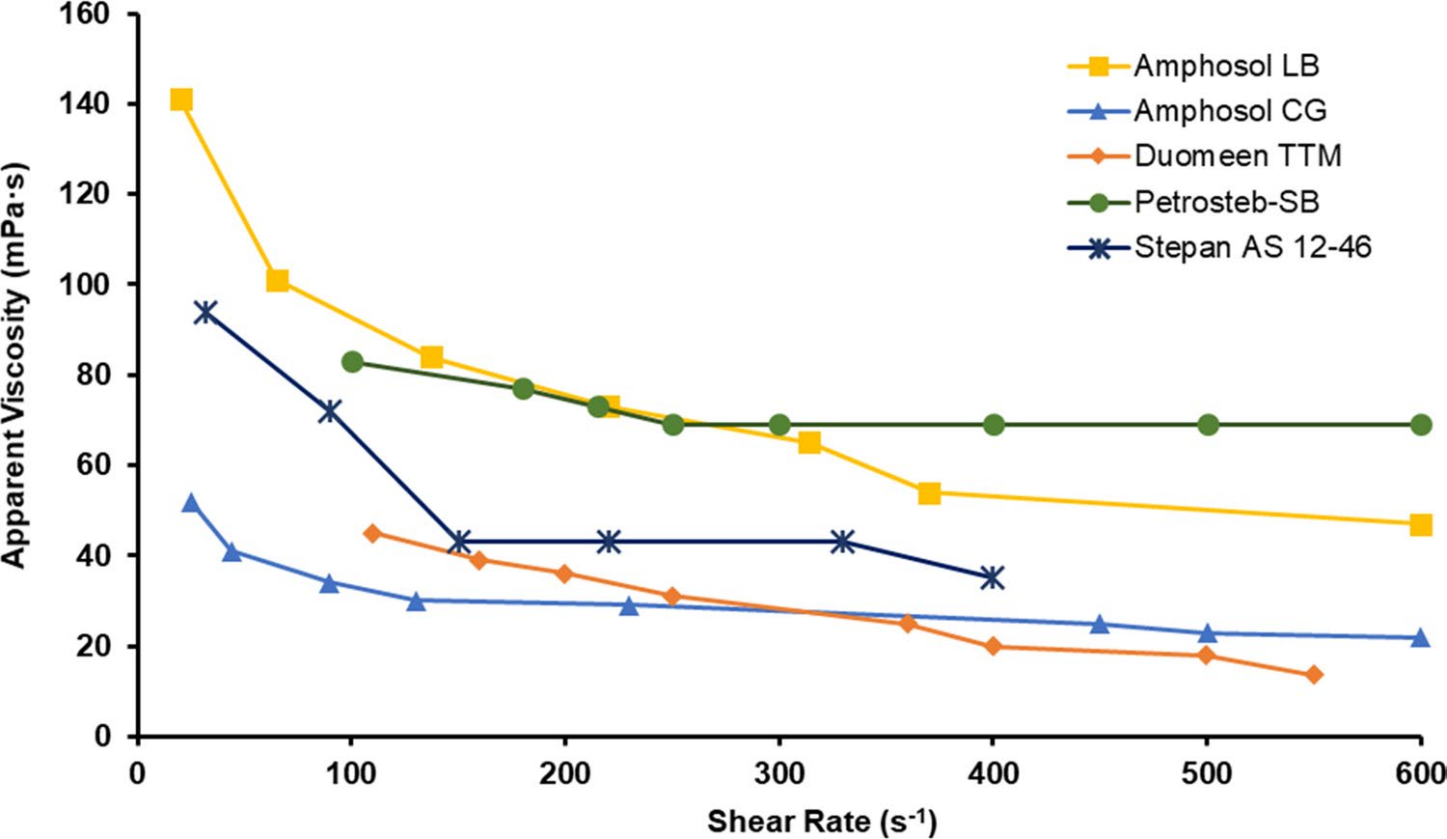
Foam apparent viscosity for 0.5 wt% Petrostep SB, Amphosol LB, Amphosol CG-50, Duomeen TTM, and Stepan AS 12-46 surfactant solutions in Brine 1 at 70% foam quality, 12 MPa, and 90 °C.

The foam rheology measurements were conducted for Petrostep SB, Amphosol LB, Amphosol-CG-50, Duomeen TTM, and Stepan AS 12-46 at 0.5 wt% surfactant solutions in Brine 1. The results, as plotted in Fig. 12, show that Petrostep SB and Amphosol LB surfactant solutions produced foams with the highest apparent viscosity. The foam viscosities for both surfactant solutions were similar up to a shear rate of 300/s, beyond which the Petrostep SB solution demonstrated a slightly higher viscosity than that of the Amphosol LB surfactant solution. The Amphosol CG-50 and Duomeen TTM surfactant solutions showed smaller foam apparent viscosities compared to the other three formulations. The foam viscosities of both the Amphosol CG-50 and Duomeen TTM surfactant solutions were almost the same across the entire range of share rates.

The foam loop rheometer and the microfluidic reservoir analogue device results are consistent in demonstrating the better performance of Amphosol LB compared to other candidate formulations. Petrostep SB on the other hand, showed higher apparent viscosity when measured using the foam loop rheometer and lower when the microfluidic device was used to compare performance among all the surfactant candidates. These results highlight the importance of the porous structure and the role of foam-wall interactions in assessing the foam quality and transport.

Overall, a combination of the microfluidic and bulk test results suggest that Amphosol LB, Duomeen TTM, and Petrostep SB are the top performers to be tested in actual core plug samples using a core flooding instrument. The Amphosol LB formulation has exhibited the highest apparent viscosity when tested using both microfluidic analogue and foam loop rheometer along with the longest foam half-life in microchannels. Duomeen TTM demonstrated good performance through the microfluidic testing and Petrostep SB showed favorable viscosity results when measured using a foam loop rheometer.

## Conclusions

Rapid microfluidic testing identified top-performing surfactant formulations for apparent viscosity enhancement and stability of supercritical CO_2_ foams. Those identified agree with conventional rheology measurement results. The microfluidic-based approach presented in this paper offers a significant advancement in the primary screening of foam additives, and time and cost savings when evaluating a large number of formulations for carbon sequestration or EOR operations. Unlike the bulk phase testing, it also enables more representative porescale foam stability analysis at relevant operating conditions. The method is versatile and can be employed for different types of gas at high-temperature high-pressure conditions, and under a wide range of pore confinements representing different reservoirs with diverse rock geometries. The method generates high-throughput data with much shorter measurement times (hours vs. days or weeks) and smaller volumes of sample (few mL vs. hundreds of mL), leading to safer and more cost-effective operation. In addition, the visualized pore confinement also enables pore-scale fluid behavior analysis and direct observation of formation damage mechanisms by precipitation and deposition of solids.

## References

[1] Max, M. D. & Johnson, A. H. Energy Overview: Energy Options and Prospects for Natural Gas. in *Exploration and Production of Oceanic Natural Gas Hydrate* 1–55 (Springer International Publishing, 2019). 10.1007/978-3-030-00401-9_1.

[2] Oei P-Y, von Hirschhausen C, Gerbaulet C, Kemfert C, Lorenz C, Oei P-Y (2018). Greenhouse gas emission reductions and the phasing-out of coal in Germany. Energiewende ‘Made in Germany’.

[3] Pedraza, J.M. The current situation and perspectives on the use of renewable energy sources for electricity generation. in *Electrical Energy Generation in Europe* 55–92 (Springer International Publishing, 2015). 10.1007/978-3-319-16083-2_2.

[4] Oam LS, Subic A, Wellnitz J, Leary M, Koopmans L (2012). Reducing emissions associated with electric vehicles. Sustainable Automotive Technologies 2012.

[5] Ajanovic A (2015). The future of electric vehicles: Prospects and impediments: The future of electric vehicles. Wiley Interdiscip. Rev. Energy Environ..

[6] Tian W, da Costa P, da Costa P, Attias D (2018). A prospective analysis of CO_2_ emissions for electric vehicles and the energy sectors in China, France and the US (2010–2050). Towards a Sustainable Economy.

[7] Koytsoumpa EI, Bergins C, Kakaras E (2018). The CO_2_ economy: Review of CO_2_ capture and reuse technologies. J. Supercrit. Fluids.

[8] Leung DYC, Caramanna G, Maroto-Valer MM (2014). An overview of current status of carbon dioxide capture and storage technologies. Renew. Sustain. Energy Rev..

[9] Artz J (2018). Sustainable conversion of carbon dioxide: An integrated review of catalysis and life cycle assessment. Chem. Rev..

[10] Nitopi S (2019). Progress and perspectives of electrochemical CO_2_ Reduction on copper in aqueous electrolyte. Chem. Rev..

[11] Shukla R, Ranjith P, Haque A, Choi X (2010). A review of studies on CO_2_ sequestration and caprock integrity. Fuel.

[12] Ajayi T, Gomes JS, Bera A (2019). A review of CO_2_ storage in geological formations emphasizing modeling, monitoring and capacity estimation approaches. Petrol. Sci..

[13] Núñez-López, V. & Moskal, E. Potential of CO_2_-EOR for near-term decarbonization. *Front. Clim.* **1**, 5 (2019).

[14] Talebian SH, Masoudi R, Tan IM, Zitha PLJ (2014). Foam assisted CO_2_-EOR: A review of concept, challenges, and future prospects. J. Petrol. Sci. Eng..

[15] Enick RM, Olsen DK, Ammer JR, Schuller W (2012). Mobility and Conformance Control for CO_2_ EOR Via Thickeners, Foams, and Gels—A Literature Review of 40 Years of Research and Pilot Tests.

[16] Zaberi HA (2020). An experimental feasibility study on the use of CO_2_-soluble polyfluoroacrylates for CO_2_ mobility and conformance control applications. J. Petrol. Sci. Eng..

[17] Guo F, Aryana S (2016). An experimental investigation of nanoparticle-stabilized CO_2_ foam used in enhanced oil recovery. Fuel.

[18] Xue Z, Panthi K, Fei Y, Johnston KP, Mohanty KK (2015). CO_2_-soluble ionic surfactants and CO_2_ foams for high-temperature and high-salinity sandstone reservoirs. Energy Fuels.

[19] Cui L (2016). Mobility of ethomeen C12 and carbon dioxide (CO_2_) foam at high temperature/high salinity and in carbonate cores. SPE J..

[20] Liontas R, Ma K, Hirasaki GJ, Biswal SL (2013). Neighbor-induced bubble pinch-off: Novel mechanisms of in situ foam generation in microfluidic channels. Soft Matter.

[21] Elhag AS (2018). Viscoelastic diamine surfactant for stable carbon dioxide/water foams over a wide range in salinity and temperature. J. Colloid Interface Sci..

[22] Convery N, Gadegaard N (2019). 30 years of microfluidics. Micro Nano Eng..

[23] Sinton D (2014). Energy: The microfluidic frontier. Lab Chip.

[24] Abolhasani M, Günther A, Kumacheva E (2014). Microfluidic studies of carbon dioxide. Angew. Chem..

[25] Gogoi S, Gogoi SB (2019). Review on microfluidic studies for EOR application. J. Petrol. Explor. Prod. Technol..

[26] Tabeling P, Chen S (2010). Introduction to Microfluidics.

[27] Anbari A (2018). Microfluidic model porous media: Fabrication and applications. Small.

[28] Zhang K, Jia N, Li S, Liu L (2019). Static and dynamic behavior of CO_2_ enhanced oil recovery in shale reservoirs: Experimental nanofluidics and theoretical models with dual-scale nanopores. Appl. Energy.

[29] Fu T, Yu X-Y (2016). Microfluidics in CO_2_ capture, sequestration, and applications. Advances in Microfluidics—New Applications in Biology, Energy, and Materials Sciences.

[30] Conn CA, Ma K, Hirasaki GJ, Biswal SL (2014). Visualizing oil displacement with foam in a microfluidic device with permeability contrast. Lab Chip.

[31] Xiao S (2018). Destabilization, propagation, and generation of surfactant-stabilized foam during crude oil displacement in heterogeneous model porous media. Langmuir.

[32] Sharbatian A, Abedini A, Qi Z, Sinton D (2018). Full characterization of CO_2_-oil properties on-chip: Solubility, diffusivity, extraction pressure, miscibility, and contact angle. Anal. Chem..

[33] Lu X, Leung DYC, Wang H, Xuan J (2018). Microfluidics-based pH-differential reactor for CO_2_ utilization: A mathematical study. Appl. Energy.

[34] Zhong, J., Abedini, A., Xua, L., Xu, Y., Qi, Z., Mostowfi, F. & Sinton, D. Nanomodel visualization of fluid injections in tight formations. *Nanoscale* **10**, 21994–22002 (2018). 10.1039/c8nr06937a 30452051

[35] Jatukaran, A., Zhong, J., Abedini, A., Sharbatian, A., Zhao, Y., Jin, Z., Mostowfi, F. & Sinton, D. Natural gas vaporization in a nanoscale throat connected model of shale: Multi-scale, Multi-component and Multi-phase. *Lab Chip* **19**, 272–280 (2019). 10.1039/c8lc01053f 30565619

[36] Xu, L., Abedini, A., Qi, Z., Kim, M., Guerrero, A. & Sinton, D. Pore-scale analysis of steam-solvent coinjection: azeotropic temperature, dilution and asphaltene deposition. *Fuel* **220**, 151–158 (2018).

[37] Wang W, Chang S, Gizzatov A (2017). Toward reservoir-on-a-chip: Fabricating reservoir micromodels by in situ growing calcium carbonate nanocrystals in microfluidic channels. ACS Appl. Mater. Interfaces.

[38] Marre S, Adamo A, Basak S, Aymonier C, Jensen KF (2010). Design and Packaging of Microreactors for High Pressure and High Temperature Applications. Ind. Eng. Chem. Res..

[39] Bao B, Sanders A, Ren G, de Haas T (2017). Rapid Microfluidic Analysis of Thermal Foam Stability at 250 °C.

[40] Nguyen P, Carey JW, Viswanathan HS, Porter M (2018). Effectiveness of supercritical-CO_2_ and N_2_ huff-and-puff methods of enhanced oil recovery in shale fracture networks using microfluidic experiments. Appl. Energy.

[41] Sayegh SG, Fisher DB (2008). Enhanced Oil Recovery by CO_2_ Flooding in Homogeneous and Heterogeneous 2D Micromodels.

[42] Zhang L (2019). Static adsorption of a switchable diamine surfactant on natural and synthetic minerals for high-salinity carbonate reservoirs. Colloids Surf. A.

[43] Fu C, Liu N (2021). Rheology and stability of nanoparticle-stabilized CO_2_ foam under reservoir conditions. J. Petrol. Sci. Eng..

[44] Da C (2018). Carbon dioxide/water foams stabilized with a zwitterionic surfactant at temperatures up to 150 °C in high salinity brine. J. Petrol. Sci. Eng..

[45] Chen, H., Cox, J. R., Ow, H., Shi, R. & Panagiotopoulos, A. Z. Hydration repulsion between carbohydrate surfaces mediated by temperature and specific ions. *Sci. Rep.* **6**, Article 28553, 1–10 (2016). 10.1038/srep28553 PMC4917866 27334145

[46] Zuta J, Fjelde I, Berenblyum R (2010). Experimental and Simulation of CO_2_-Foam Flooding In Fractured Chalk Rock at Reservoir Conditions: Effect of Mode of Injection on Oil Recovery.

[47] Zhang Y (2015). CO_2_ foam flooding for improved oil recovery: Reservoir simulation models and influencing factors. J. Petrol. Sci. Eng..

[48] Gizzatov A (2019). Nanofluid of petroleum sulfonate nanocapsules for enhanced oil recovery in high-temperature and high-salinity reservoirs. Energy Fuels.

[49] Farzaneh SA, Sohrabi M (2015). Experimental investigation of CO_2_-foam stability improvement by alkaline in the presence of crude oil. Chem. Eng. Res. Des..

[50] Wang Y (2017). The stability study of CO_2_ foams at high pressure and high temperature. J. Petrol. Sci. Eng..

[51] Guiñón, J., Ortega, E., García-Antón, J. & Pérez-Herranz, V. *Moving average and Savitzki–Golay smoothing filters using mathcad.* (International Conference on Engineering Education, Coimbra, 2007).

